# *About the Cover* Vincent van Gogh (1853–1890). The Prison Courtyard (1890)

**DOI:** 10.3201/eid0909.AC0909

**Published:** 2003-09

**Authors:** Polyxeni Potter

**Affiliations:** * Centers for Disease Control and Prevention, Atlanta, Georgia, USA

**Figure:**
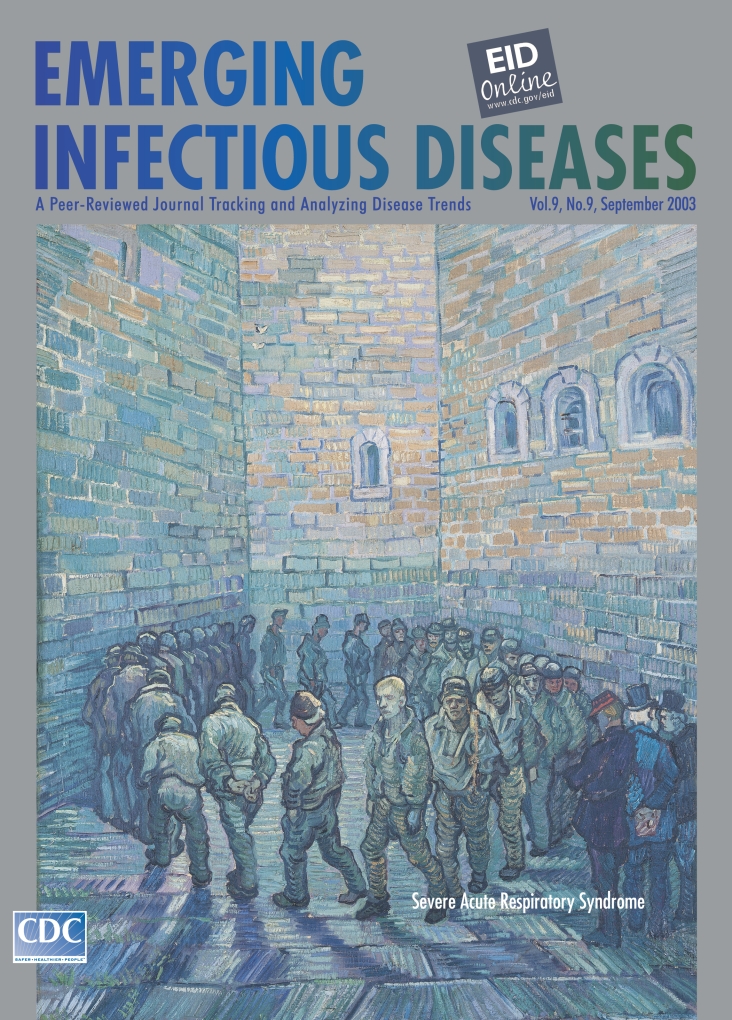
**Vincent van Gogh (1853–1890). The Prison Courtyard (1890)** Oil on canvas, 80 cm x 64 cm. The State Pushkin Museum of Fine Arts, Moscow, Russia

Walls (1896)Without consideration, without pity, without shame they have built big and high walls around me.And now I sit here despairing. I think of nothing else: this fate gnaws at my mind;for I had many things to do outside.  Ah why didn’t I observe them when they were building the walls?But I never heard the noise or the sound of the builders. Imperceptibly they shut me out of the world.

From The Complete Poems of Cavafy, copyright 1961, renewed 1989 by Rae Dalven, reprinted by permission of Harcourt, Inc.

Van Gogh painted The Prison Courtyard while “imprisoned” himself, in the Saint-Paul-de-Mausole asylum in Saint Rémy. He died 5 months later of a self-inflicted gunshot wound, the culmination of his long struggle with physical and mental illness (*1*).

The first Dutch master since the 17th century, van Gogh did not become an artist until 10 years before the end of his life. His early interests were literature and theology. The son of a protestant minister, he had a strong sense of mission, which was reinforced by his dislike of industrial society and his work as lay preacher among poor coal miners in Belgium. That experience with human misery influenced his art, which had an air of mysticism and austerity and, like that of his contemporary Honoré Daumier, often featured the oppressed and the downtrodden (e.g., The Potato Eaters, 1885) (*2*).

Van Gogh was influenced by Degas, Seurat, and other leading French artists. His use of pure color to define content places him ahead of his contemporaries as a forerunner of expressionism. “I will paint with red and with green the terrible passions of humanity,” he wrote. “…Instead of trying to reproduce exactly what I see before my eyes, I use color more arbitrarily to express myself forcibly.” As if he knew that his artistic career would be very brief, van Gogh painted with a sense of urgency, almost scarring the canvas with thick overlaid brush strokes that were distinct, deliberate, and intense. He wanted “to exaggerate the essential and to leave the obvious vague” (*3*,*2*). His paintings became vehicles of his emotional condition, shifting like his moods from the brightest landscapes (Sunflowers, Irises) to the darkest manifestations of personal symbolism (Starry Night, Wheat Field with Crows).

Van Gogh created his best work between 1888 and 1890, when he went to Arles in the south of France (*3*). There, in the Mediterranean countryside, he painted landscapes of almost pulsating light, as he tried to convey the spiritual meaning he believed animated all things. Exaggerating his figures with vibrant hues, he set them against thick, rough circles like halos, freeing them from static background and hurling them into infinity. Wishing to form an artists’ colony in the region, he invited Paul Gauguin to join him “in this kingdom of light.” The brief collaboration (less than 2 months) produced many noted works but ended abruptly when van Gogh became violent, attacked his friend with a straight razor, then in remorse cut off his own ear and offered it to a local prostitute.

Gauguin departed for Tahiti, while van Gogh, his health in a downward spiral, entered the asylum in Saint Rémy, where with the approval of his doctors he continued to paint. No one knows the cause of van Gogh’s angst. His seizures, hallucinations, violent mood swings, and increasing anxiety have been variously diagnosed as neurologic disorder, depression, alcoholism, venereal disease, chemical or metabolic imbalance, and behavioral disorder possibly caused by a virus (*4*). While illness largely defined what the artist could and could not do (he only painted when he was lucid), art gave him reason to continue living. Even in confinement, his work extended far beyond his personal circumstance.

The Prison Courtyard on this month’s cover of Emerging Infectious Diseases expresses the artist’s hopelessness and despair. In the lower part of the painting, thirty-three inmates form a human corona, pacing heads down, in defeated rote and joyless resignation. In spite of the shared misery and monochrome prison garb, they are not uniformly anonymous; some faces can be deciphered, particularly the one in the center, whose blond hair is lighted by an imperceptible sun’s ray. That is van Gogh himself in what has been interpreted as a “metaphoric self-portrait” (*1*,*5*).

Merged with the pavement, the prison walls loom high above the inmates’ heads, overpowering the canvas with finality and forcefulness. The harsh, impenetrable structure, so devoid of beauty, encages the inmates outside the common web of human interaction. These literal walls painted by van Gogh “in captivity” allude to the harsher metaphorical walls of his unknown illness and his spiritual isolation.

The causes of aberrant behavior that leads to imprisonment are largely unknown, as are the causes of many diseases and their consequent spiritual isolation. When an old microbe, a coronavirus, causes a new disease, severe acute respiratory syndrome, the unknown nature of the disease and the risk for contagion require containment to arrest spread of infection. To prevent and control physical illness, the exposed and the infected (in the case of SARS many healthcare workers) may also have to face spiritual isolation.
